# The Piezoresponse in WO_3_ Thin Films Due to N_2_-Filled Nanovoids Enrichment by Atom Probe Tomography

**DOI:** 10.3390/ma16041387

**Published:** 2023-02-07

**Authors:** Pamela M. Pineda-Domínguez, Torben Boll, John Nogan, Martin Heilmaier, Abel Hurtado-Macías, Manuel Ramos

**Affiliations:** 1 Departamento de Física y Matemáticas, Instituto de Ingeniería y Tecnología, Universidad Autónoma de Ciudad Juárez, Avenida del Charro 450 N, Cd. Juárez, Chihuahua 32310, Mexico; 2 Institut für Angewandte Materialien-Werkstoffkunde (IAM-WK), Karlsruhe Institute of Technology, Engelbert-Arnold-Strasse 4, 76131 Karlsruhe, Germany; 3 Karlsruhe Nano Micro Facility (KNMFi), Karlsruhe Institute of Technology (KIT), Hermann-von-Helmholtz-Platz 1, 76344 Eggenstein-Leopoldshafen, Germany; 4 Institute for Nanotechnology (INT), Karlsruhe Institute of Technology (KIT), Hermann-von-Helmholtz-Platz 1, 76344 Eggenstein-Leopoldshafen, Germany; 5 Center for Integrated Nanotechnologies, 1101 Eubank Bldg. SE, Albuquerque, NM 87110, USA; 6 Laboratorio Nacional de Nanotecnología, Centro de Investigación en Materiales Avanzados S.C., Miguel de Cervantes 120, Complejo Industrial Chihuahua, Chihuahua 31109, Mexico

**Keywords:** WO_3_, films, PFM, APT, nanovoids

## Abstract

Tungsten trioxide (WO_3_) is a versatile *n*-type semiconductor with outstanding chromogenic properties highly used to fabricate sensors and electrochromic devices. We present a comprehensive experimental study related to piezoresponse with piezoelectric coefficient *d*_33_ = 35 pmV^−1^ on WO_3_ thin films ~200 nm deposited using RF-sputtering onto alumina (Al_2_O_3_) substrate with post-deposit annealing treatment of 400 °C in a 3% H_2_/N_2_-forming gas environment. X-ray diffraction (XRD) confirms a mixture of orthorhombic and tetragonal phases of WO_3_ with domains with different polarization orientations and hysteresis behavior as observed by piezoresponse force microscopy (PFM). Furthermore, using atom probe tomography (APT), the microstructure reveals the formation of N_2_-filled nanovoids that acts as strain centers producing a local deformation of the WO_3_ lattice into a non-centrosymmetric structure, which is related to piezoresponse observations.

## 1. Introduction

Tungsten trioxide (WO_3_) is an *n*-type semiconductor with chromogenic and catalytic properties that has been used as an electrochromic layer for smart windows [1,2], gas sensors [3,4], and water-splitting devices [5,6,7]. A piezoelectric behavior of WO_3_ would open an opportunity for diverse applications in the field of the Internet of Things (IoT) [8,9] and micro-electromechanical systems (MEMS) [10,11]. The piezoelectric effect has been explained to occur only in crystal structures with non-centrosymmetric space group [12,13], which lack an inversion point, and hence, a decompensation of charges provides the formation of polarized domains. The piezoelectric property is usually found in ceramic materials [13] and semiconductors [14], with lead zirconate titanate (PZT) as the most common material with remarkable piezoresponse derived from an interaction at the morphotropic phase boundary (MPB) [15,16]. The piezoelectric effect is usually measured by piezoresponse force microscopy (PFM), through the estimation of the piezoelectric coefficient *d*_33_, the obtention of hysteresis loops and switching imaging of the topography showing piezo-active domains [17,18]. However, non-piezoelectric effects [10,18,19,20,21,22,23,24] can induce electromechanical (EM) responses in PFM measurements that resemble the piezoresponse. These can be electrostatic effects [25,26,27], electrochemical strain [28,29,30,31], induced polarization (electrostriction) [32], flexoelectricity [33,34,35,36], or thermal expansion due to Joule heating [37]. Such so-called “non-piezoelectric effects” in the literature are strongly related to ionic migration and diffusion along oxygen vacancies [38], which are considered the most common functional defects [1,38,39,40,41] in transition metal oxides, and can induce electronic structure-related properties in oxides. Li et al. described how oxygen vacancies can promote dislocations, and thus induce ion diffusion on lattice sites of WO_3_ [42]. In a non-stoichiometric WO_3-x_, those are also active sites for chemical adsorption of light molecules for gas sensing of H_2_ [3,43,44], N_2_ [45], NO_2_ [46], and NH_3_ [47,48] among other species, and are mobile defects with a high diffusion coefficient [38], which can be tuned by electric fields, temperature, pressure, or light. Park et al. observed that the rearrangement of oxygen vacancies while applying a direct electric field can break the crystallographic symmetry [49,50], inducing a large piezoelectric effect in Gd-doped CeO_2-x_, an intrinsically centrosymmetric fluorite, by electric field-induced redistribution of mobile oxygen vacancies. The formation of oxygen vacancy can produce a micro volume expansion of the films, leading to bending and to a strain–electrical field relationship. Seol et al. proved the electrochemical strain contribution to piezoresponse in non-piezoelectric TiO_2_ thin films, attributing the effect to oxygen vacancies redistribution by an applied electric field during PFM measurement [23].

Although WO_3_ is not a natural piezoelectric, there is strong evidence of piezoresponse in this material. Yun et al. found a lateral (in-plane) PFM piezoresponse for WO_3_ of *d*_33_ = 6 pmV^−^^1^ attributed to induced flexoelectric polarizations due to strain-gradients at ferroelastic domain walls of a monoclinic (P2_1_/n) structure [33]. Kim et al. found a piezoresponse measured by PFM in vertical (out-of-plane) mode in an oxygen-deficient WO_2.96_ film with a *d*_33_ coefficient of 7.9 pmV^−^^1^, attributed to the conductivity in twin walls in a non-centrosymmetric tetragonal phase [51]. This conductivity is explained by the accumulation of oxygen vacancies at the domain walls, donating free charges and increasing local charge flow near twin walls [51,52]. As well as non-centrosymmetric structures, or functional defects such as oxygen vacancies, there is also a relation between piezoelectric effect and other functional defects, such as porosity or nanovoids. Liu and Wang reported a model indicating a notable role of porosity distribution on the eigenfrequency of functionally graded piezoelectric materials (FGPM) [53]. Li et al. modeled the influence of voids in piezoelectric coefficients considering permeability and volume fraction of the voids [54].

Here, we report a piezoresponse measured by PFM in a WO_3_ film deposited on sapphire (Al_2_O_3_) by radio-frequency sputtering technique with post-deposit annealing at 400 °C under forming gas conditions. Structural and chemical characterization carried out by X-ray diffraction (XRD) and atom probe tomography (APT) reveals that our piezoresponse is related to local polar structure produced by nanovoids filled with N_2_.

## 2. Materials and Methods

The tungsten trioxide (WO_3_) thin films were deposited by radio frequency (RF) magnetron sputtering technique in a Kurt J. Lesker PVD75 equipment using a commercial target of WO_3_ (99.95%) and Al_2_O_3_ (99.9%) as substrate. The base pressure was set up to 1.3 × 10^−4^ Pa and the deposition process was run with a working pressure of 0.4 Pa and 225 W of RF power, achieving a deposition rate of 1 Å/s. As-deposited samples were annealed in a Qualiflow-Jiplec Jetfirst 100 furnace at a reduced pressure processing in a 3% H_2_/N_2_ forming gas environment (0 to 200 scm in 15 s). The temperature was increased with a ramp of 1°/s until reaching 400 °C or 500 °C, respectively, and after a dwell time of 45 min, it was cooled down with a ramp of 1°/s for 15 min. A film thickness of 220 nm was measured for the as-deposited film using an Ambios XP-2 profilometer (Supplemental Material).

The domain imaging and hysteresis loops were obtained using a Dual AC Resonance Tracking (DART) mode with Switching Spectroscopy Piezoresponse Force Microscopy (SS-PFM) using an Atomic Force Microscope (AFM) model Infinity 3D Asylum Research^®^ equipped with two internal lock-ins amplifiers. Identification of the surface domain structure was performed in vertical mode with an AC voltage amplitude of 5 V_pk-pk_ at a drive frequency of 398 kHz, far below the cantilever’s resonance, and applied between the bottom electrode and the Pt/Ir conductive tip during PFM imaging. SS-PFM obtained local polarization and hysteresis loop evaluation with an applied voltage from −10 V to 10 V. In order to diminish electrostatic effects, an electrically charged Ag landing electrode was placed near the measured area.

The crystallographic structure was studied using a Panalytical Empyrean system with a Cu Kα radiation source (λ = 1.54 Å) at an operating voltage of 40 kV and emission current of 30 mA with a scanning angle of 20° to 80° and step size of 0.05°. Structure analysis was completed using a high-resolution transmission electron microscopy (HRTEM) model JEOL^®^ JEM-2200FS+Cs equipped with a spherical aberration corrector in the condenser lens and operated at an accelerating voltage of 200 kV. Samples were prepared using a JEOL^®^ JEM-9320 focused ion beam (FIB) system operated at 30 kV (Supplemental Material).

The chemical elemental distribution was studied using a Cameca^®^ Local Electrode Atom Probe (LEAP 4000X HR) system equipped with a UV laser (λ~355 nm). For the measurements, the temperature was set to 50 K with a detection rate of 0.3%, a pulse frequency of 100 kHz, and a laser beam energy of 30 pJ. The specimens were prepared using the standard lift-out process and annular milling with focused ion-beam (FIB) [55] in a scanning electron microscope (Zeiss^®^ Auriga 60©). All data were reconstructed with Cameca AP Suite 6.1. Compositional profiles were obtained along the z-axis of the images corresponding to film growth direction, with a bin width of 0.1 nm and background corrected. 

## 3. Results and Discussion

### 3.1. Piezoresonse by PFM

**Figure 1:**
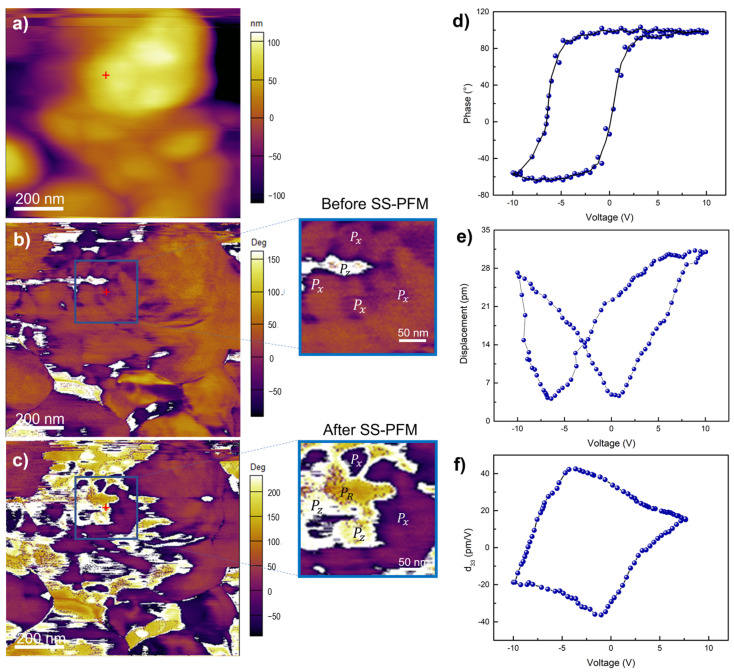
(**a**) Topography in contact mode of WO_3_ thin film annealed at 400 °C. PFM phase signal images (**b**) before and (**c**) after measurement of the local hysteresis loops revealing variation of the structure of domains with different orientation of polarization; (**d**) amplitude, (**e**) phase, and (**f**) piezoelectric coefficient (*d*_33_) versus applied bias.

By local switching and hysteresis behavior during PFM measurements, a piezoresponse was confirmed for WO_3_ films processed at 400 °C. Figure 1a shows the surface topography and grain domains. The phase signals before and after measurements, revealing the existence of domains with different polarization orientations, as described by Kholkin et al. [56,57], are presented in Figure 1b,c, respectively. Insets show the region directly affected by the SS-PFM measurement, where the reorientation of polarization with the applied electric field is observed, due to an indirect piezoelectric effect. White regions correspond to positive polarization domains (P_z_), dark violet regions correspond to polarization in-plane (P_x_) towards the bottom electrode, while yellow regions correspond to a remanent polarization; thus, an evident reorientation of polarization is achieved as found by many authors with this technique [17,18,19,58,59]. The hysteresis loop from switching polarization domains at a phase difference of 180° using AC voltage is presented in Figure 1e. The amplitude (nm) versus bias voltage (V) plot in Figure 1d exhibits a butterfly loop related to local deformation under the applied bias, which demonstrates polarization in granular domains, as described by Roelofs et al. [60]. Even with DART mode it was possible to see that loops can be shifted towards a negative applied bias, suggesting electrostatic contributions to the electromechanical response. These are commonly explained by oxygen vacancies, which produce a high surface electrostatic potential [23,25,27,61]. The local *d*_33_ coefficient in Figure 1f was estimated using the relation (V − V_1_)*d*_33_ = D − D_1_ [62,63], where D is the piezoelectric deformation or amplitude and V the applied voltage, respectively. D_1_ and V_1_ designate the values at the intersection in the butterfly loop in Figure 1d. A coercive voltage of 2.7 V was obtained by using the relation (V_c_^+^ − V_c_^−^)/2, where V_c_^+^ and V_c_^−^ are forward and reverse coercive bias voltages, respectively. A piezoelectric coefficient *d*_33_ of 35 pmV^−1^ was obtained for a maximum voltage of 10 V, which is four times higher than that reported by Kim et al. for WO_2.96_ [51]. The piezoresponse was only observed for the film annealed at 400 °C, but neither for the as-deposited nor for the 500 °C specimen (Supplemental Material).

### 3.2. Crystal Structure Analysis

**Figure 2:**
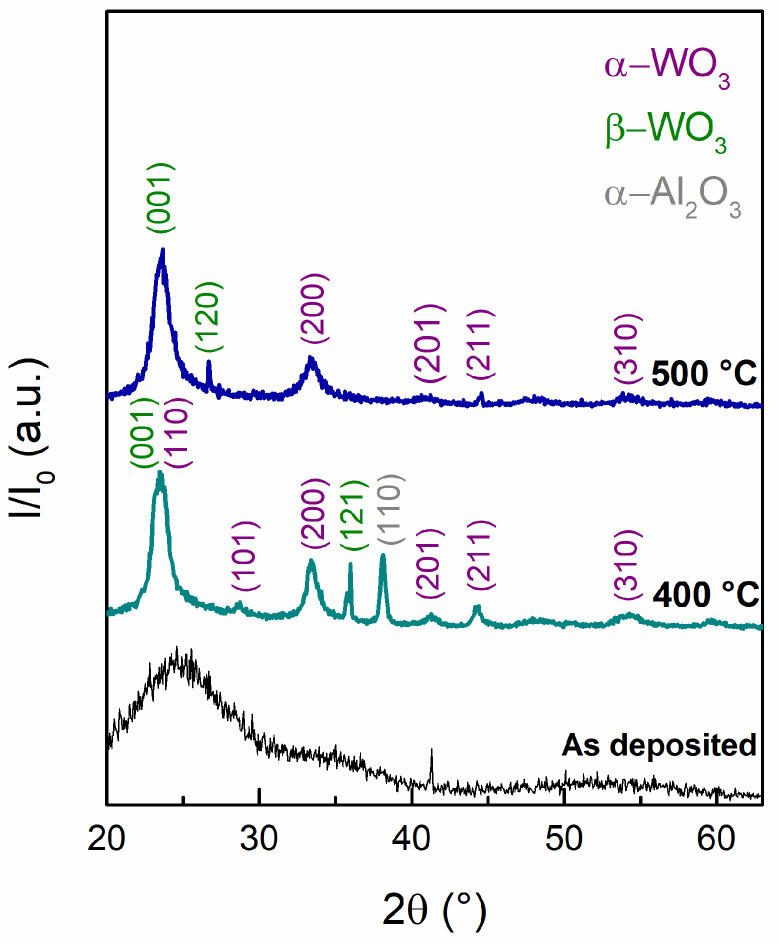
X-ray diffraction pattern for WO_3_ thin films deposited onto sapphire (Al_2_O_3_) as deposited and annealed at 400 °C and 500 °C in a 3% H_2_/N_2_ forming gas environment.

The crystallographic structures were studied using the X-ray diffraction technique; the results indicate an amorphous structure for the as-deposited film and crystalline structures for films annealed at 400 °C and 500 °C, as presented in Figure 2. It is known that WO_3_ crystal structures may form during the annealing process as follows: monoclinic (γ-WO_3_) above 300 °C, orthorhombic (β-WO_3_) between 400 °C and 600 °C, and tetragonal (α-WO_3_) above 700 °C [64,65]. The diffraction pattern of the sample showing the piezoresponse correspond to a mixture of phases; the plane diffraction at ~23° is a contribution of (001) plane of orthorhombic (PDF 00-020-1324) and (110) of tetragonal (PDF 00-018-1417) WO_3_ phases. Diffraction planes at 28.7°, 33.5°, 41.2°, 48.8°, 54.5°, and 59.7° may correspond to any of those phases.

**Figure 3:**
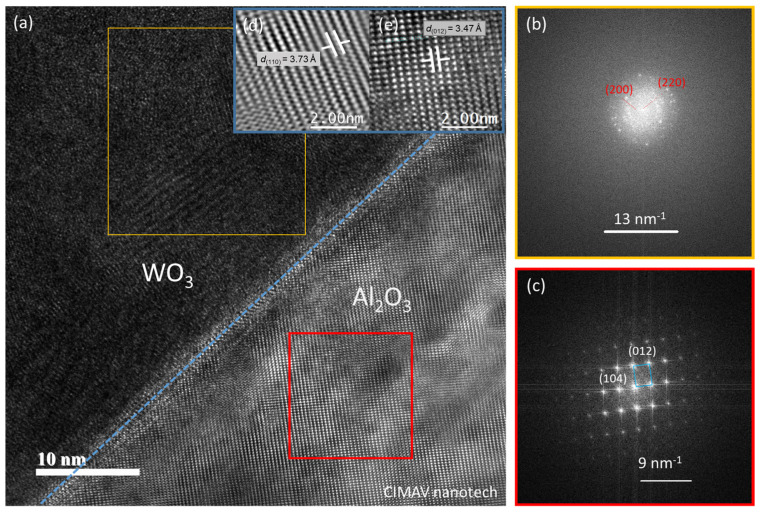
(**a**) High-resolution Cs-corrected TEM images. A selected area electron diffraction pattern of (**b**) WO_3_ thin film corresponding to a polycrystalline structure, including a tetragonal (P4/nmm) structure, and of (**c**) the sapphire substrate with monocrystal of an Rc3 structure. Atomic arrays of (**d**) film and (**e**) substrate structures, with lattice plane distances of d_(110)_ = 3.74 Å and d_(012)_ = 3.47 Å, respectively.

By using HRTEM, we were able to confirm the presence of a non-centrosymmetric α-WO_3_, as presented in Figure 3, which shows a clear interface between film and single crystal sapphire (α-Al_2_O_3_) substrate. The selected area electron diffraction (region in yellow frame) corresponds to a polycrystalline structure, which allows for distinguishing the diffraction planes (200) and (220) with an interplanar distance d_110_ = 3.74 Å of a non-centrosymmetric α-WO_3_ (P4/nmm) (PDF 01-018-1417). Diffraction planes (012) and (104) with an interplanar distance d_012_ = 3.47 Å corresponding to the sapphire (α-Al_2_O_3_) substrate with space group R-3c (167) (PDF 01-077-2135) were also identified, in agreement with previous results with the diffraction plane (110) at ~38°. The film annealed at 500 °C has a predominant contribution of an orthorhombic structure (PDF 00-020-1324), but also traces of α-WO_3_; however, no piezoresponse was found in this film, suggesting different features related to its final crystal structure.

### 3.3. Composition Distribution Analysis by APT

Mass spectra corresponding to time-of-flight events were analyzed to achieve volume rendering of the as-deposited samples and those annealed at 400 °C and 500 °C. The mass spectra show a significant difference in the range of mass-to-charge ratios corresponding to N-species and Ar between the analyzed samples (Supplemental Material). The overlapping peaks at 28 Da in the mass spectra can lead to wrong interpretations since it can be attributed to AlH or N_2_. In this case, the presence of AlH was excluded because the Al^3+^ is missing in the spectrum. The as-deposited sample mass spectrum presents a low intensity of peaks attributed to N_2_, which increase significantly at 400 °C of annealing treatment under forming gas and decrease at 500 °C. A significant amount of Ar is present in the as-deposited sample, with a peak at 41 Da, which we attribute to ArH^−^. The Ar concentration decreases significantly in the sample annealed at 400 °C, and increases at 500 °C.

**Figure 4:**
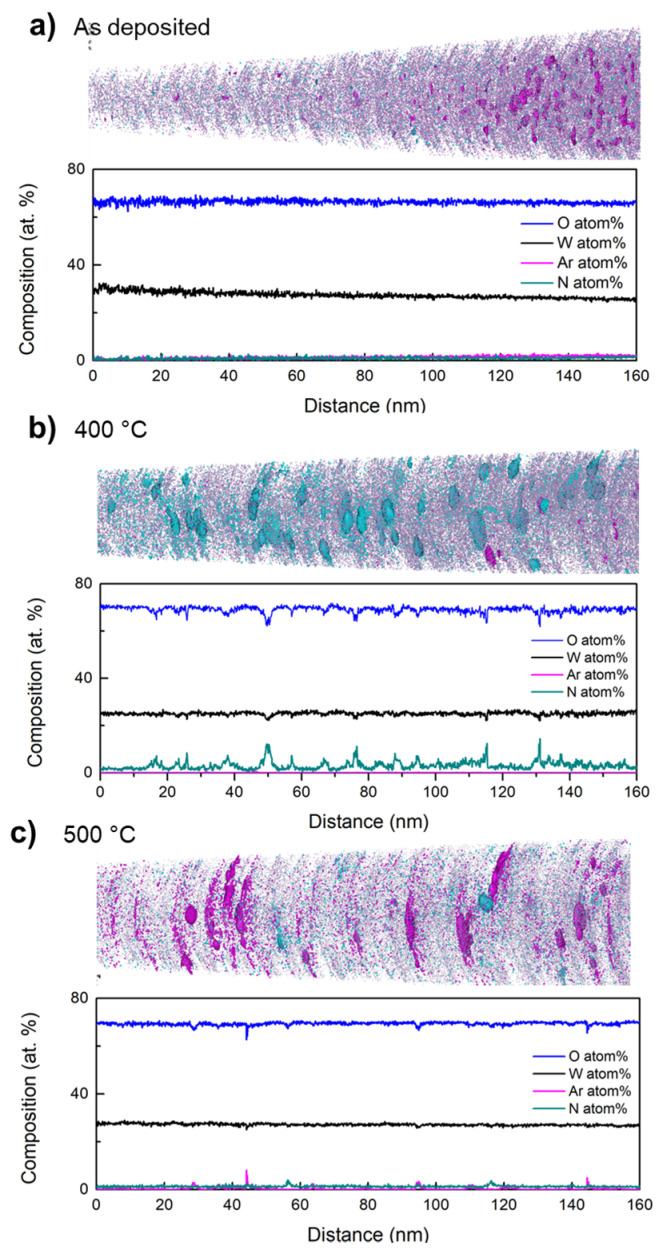
Atom probe tomographs (up) with corresponding concentration profiles (down) for (**a**) as deposited and annealed at (**b**) 400 °C and (**c**) 500 °C WO_3_ thin films deposited onto Al_2_O_3_.

The peak identification in each mass spectra allows us to obtain the chemical volume distributions and composition profile of the specimens. Tomographies of the whole FIB-needled tip reveal defined interfaces, meaning strong adherence between WO_3_ and the α-Al_2_O_3_ ([0001] sapphire) substrate. The analysis ahead was performed for reconstructions of 160 nm of the tips, at 20 nm far from the sapphire, as presented in Figure 4, with WO_3_ ions and isosurfaces for Ar and N_2_ ions. Composition profiles elucidate the atomic composition shown in Table 1, supporting the observations in the mass spectra for Ar and N_2_.

**Figure 5:**
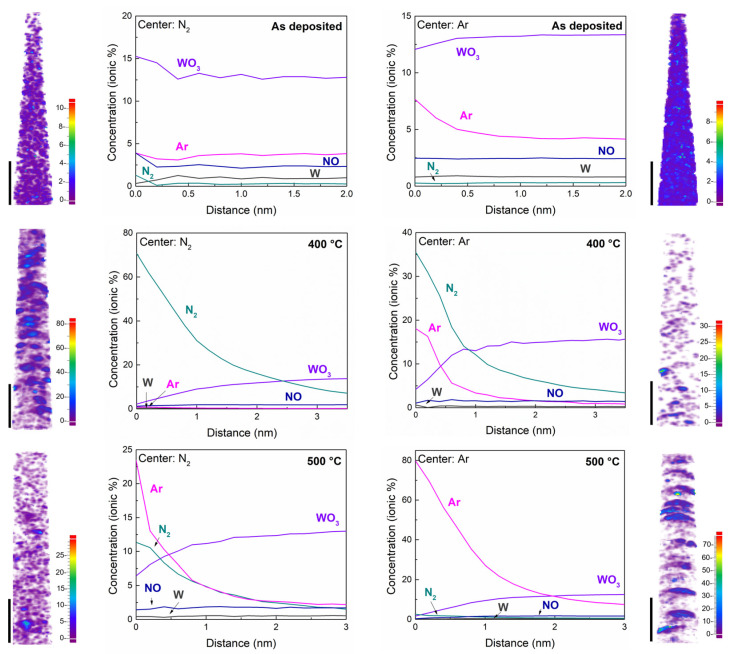
Radial distribution function (RDF) and heatmaps for cluster analysis of Ar and N_2_ in WO_3_ thin films as deposited and annealed at 400 °C and 500 °C in a forming gas (3% H_2_/N_2_).

To characterize the spatial environment of ions the radial distribution function (RDF) and nearest neighbor distribution (NND) (Supplemental Material) were applied. RDF describe how many neighboring ions of a particular ion type have a specific direction and radial distance, while NND gives the distribution of distances between ions of the same type and their individual nearest neighbor [66]. In order to identify chemical compounds that might be clustering, RDF was obtained for several atom centers, such as Ar, W-species (W, WO, WO_2_, W_2_O_5_, W_2_O), and N-species (N_2_, NO, N_2_O, NO_2_). Except for Ar and N_2_, RDF do not show good qualities for any other center ion to proceed further in the clustering analysis, either because the compounds are found in a very low atomic percentage or because the distribution is homogeneous. RFD for the as-deposited sample (Figure 5a) reveal some clustering distribution of Ar with a small gradient towards the interface with the sapphire, suggesting the formation of defects, i.e., oxygen vacancies, during the deposition process. At this stage, N-species are homogeneously distributed in the film matrix.

For the samples annealed at 400 °C and 500 °C, clustered platelet-shaped regions with high N_2_ or Ar content were observed in the reconstructions, suggesting the formation of nanovoids filled with these atomic species and “spilled out” during evaporation in the APT measurement. The nanovoids in the 400 °C sample showing the piezoresponse consist mainly of N_2_. It is expected that the annealing process induces crystallization, and also the formation of oxygen vacancies [40,64,67], which can act as active sites for the chemical adsorption of N molecules [45,68,69]. At 400 °C the Ar is released from the film leaving behind nanovoid distribution inside the polycrystalline WO_3_ matrix, allowing N_2_ trapping during the annealing treatment in a forming gas environment. Fewer nanovoids were observed in the sample processed at 500 °C (Figure 5c), filled predominantly with Ar. In this specimen it was determined that the N-concentration is homogeneously distributed as N-species (NO, N_2_O, NO_2_), proving the temperature-dependence of the action of the forming gas, as it has been observed in tin oxide layers [70]. RDF shows some Ar clustering, which suggests that it must be a competition of reactions in which the formation of N-species is preventing the Ar from becoming trapped in the nanovoids. The non-centrosymmetric structure (α-WO_3_) was also found in the films without piezoresponse; hence, the presence of a non-centrosymmetric structure is not sufficient in this material to achieve piezo-active domains detected by PFM. However, nanovoids observed by APT may be indirectly related to the piezoresponse. Such nanovoids could act as strain centers promoting a local break symmetry, and thus, the defect-induced polarity related with the piezoresponse measured by PFM.

## 4. Conclusions

Fabrication of tungsten trioxide WO_3_ thin films using RF-sputtering over sapphire substrates with post-deposit annealing processing at 400 °C and 500 °C in a forming gas environment were studied by piezo-force microscopy (PFM), obtaining a piezoresponse with *d*_33_ = 35 pmV^−1^ by domain piezoresponse imaging and piezoelectric hysteresis loops in thin films processed at 400 °C. The piezoresponse is mainly attributed to a local break in the symmetry related to N_2_-filled nanovoid distribution as revealed by atom probe tomography. Complementary characterization by electron microscopy in scanning and transmission mode and X-ray diffraction, indicates a mixture of orthorhombic and tetragonal phases for samples processed at 400 °C.

## Figures and Tables

**Table 1:** Atomic composition of O, W, Ar, and N_2_ in WO_3_ thin films as deposited and annealed at 400 °C and 500 °C.

	As Deposited	400 °C	500 °C
O	66.2	69.4	71.3
W	27.1	25.2	27.4
Ar	1.9	0.04	0.4
N	1.2	3.1	0.8

## Data Availability

No data are shared.

## Supplementary Materials

The following supporting information can be downloaded at https://www.mdpi.com/article/10.3390/ma16041387/s1, Figure S1: Mass spectra. Figure S2: Radial distribution function. Figure S3: Nearest neighbor distribution. Figure S4: Annealing profile. Figure S5: Profilometry. Figure S6: PFM off field for WO_3_ at 500 °C. Figure S7: EDS and SEM for WO_3_ thin film. Figure S8: Cross-sectional SEM image of WO_3_. Figure S9: Lamella preparation for TEM. Figure S10: Mass spectra of WO_3_ thin films measured by APT.
