# Supplementary material for: The Piezoresponse in WO3 Thin Films Due to N2-Filled Nanovoids Enrichment by Atom Probe Tomography
Source: Materials (Basel). 2023 Feb 7;16(4):1387. doi: 10.3390/ma16041387 (PMC9960742; doi:10.3390/ma16041387)
Supplement: Supplementary file 1 [file materials-16-01387-s001.zip › materials-2190617-supplementary.pdf]

The piezoresponse in WO_3 thin films due to N_2-filled nanovoids enrichment by Atom Probe Tomography

Supplemental Material 1

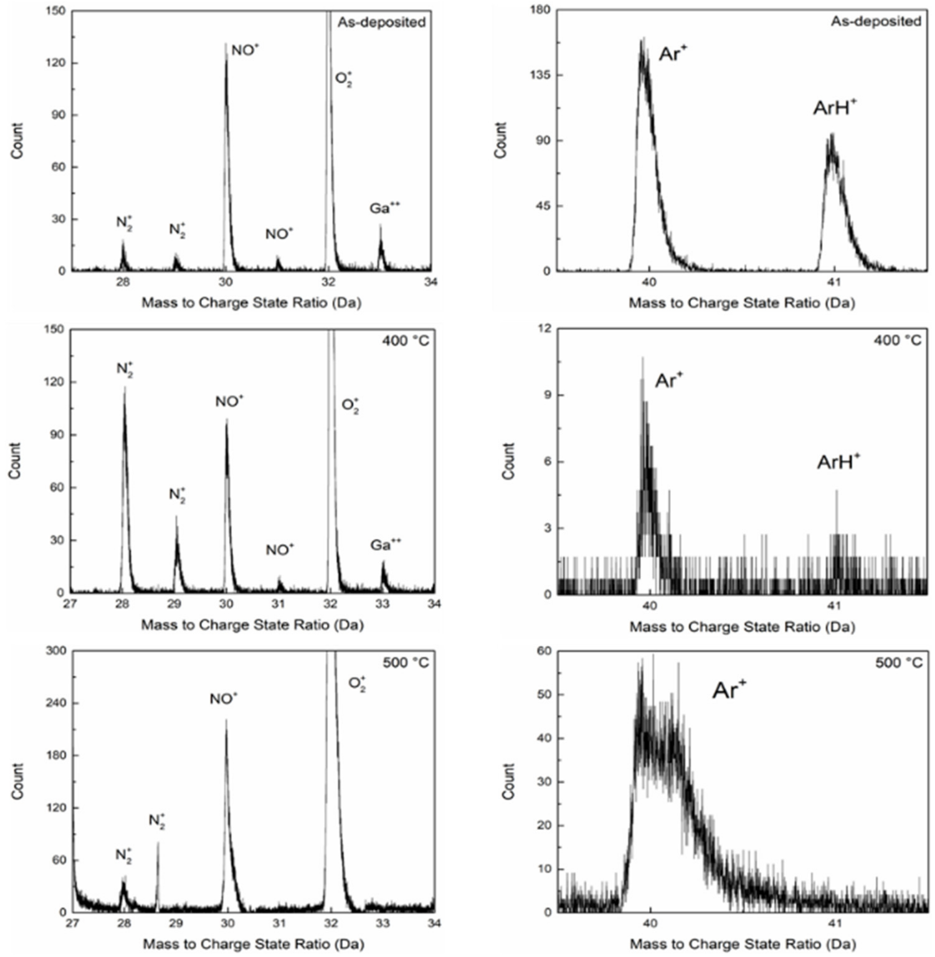

Figure S1. Mass spectra showing Ar and N-species content in as deposited, annealed at 400 °C and 500 °C WO_3 thin films.

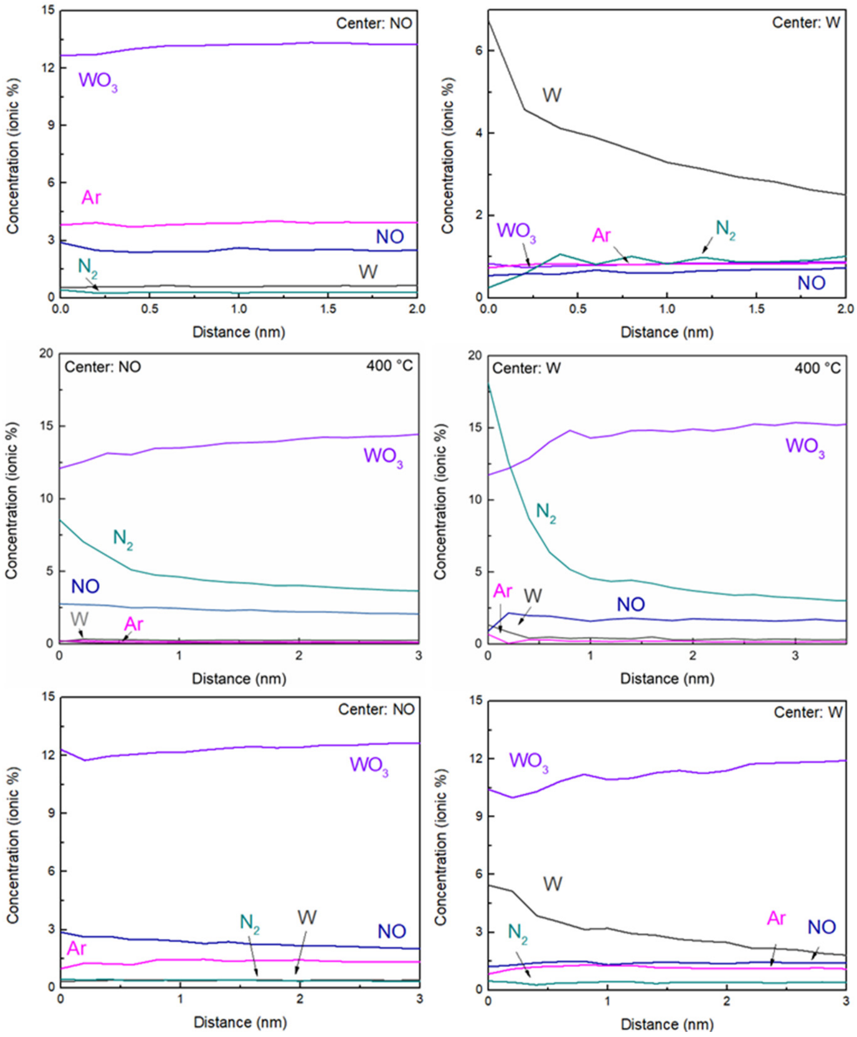

Figure S2. Radial distribution function with NO and W center ions.

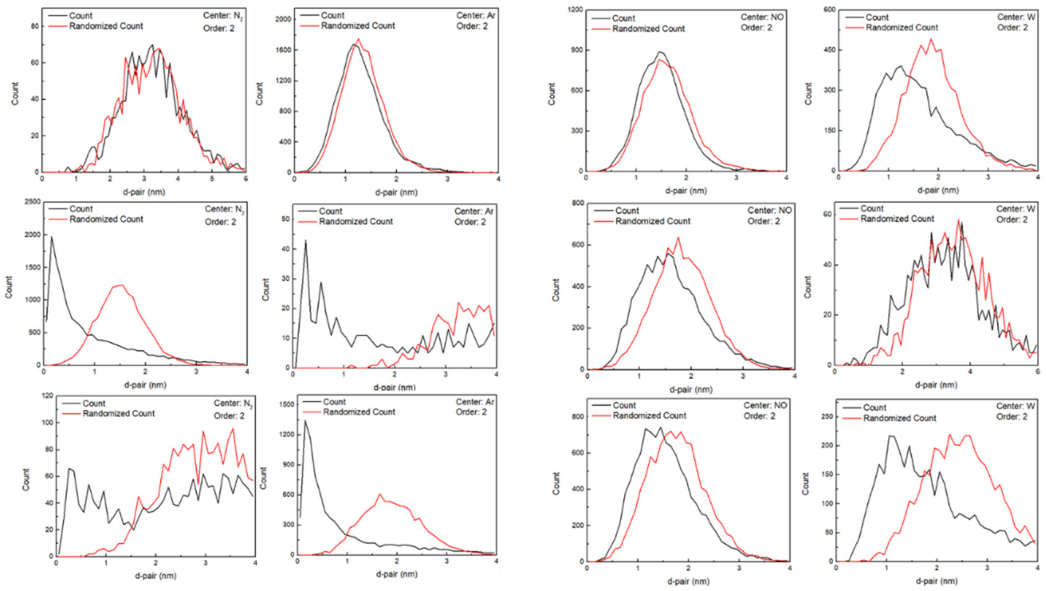

Figure S3. Nearest neighbor distribution for WO_3 thin films as deposited, annealed at 400 °C and 500 °C.

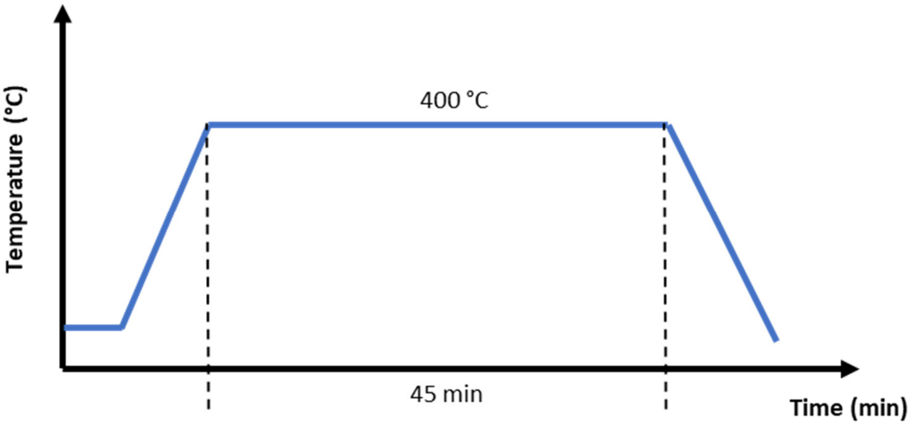

Figure S4. Annealing profile used for WO_3 thin film processing.

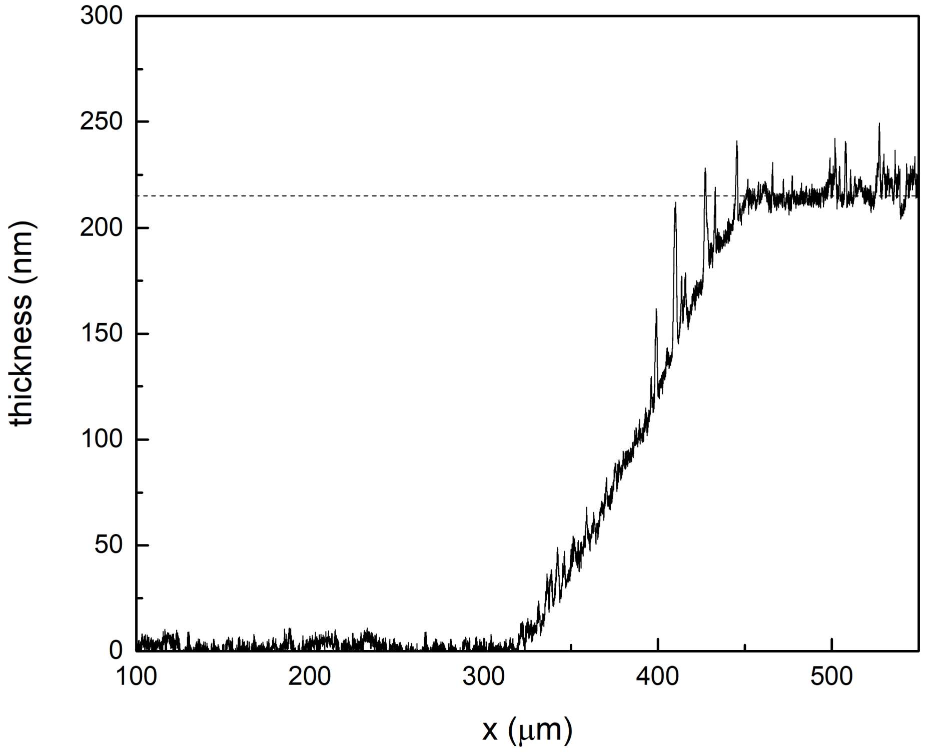

Figure S5. Profilometry to measure film thickness of WO_3.

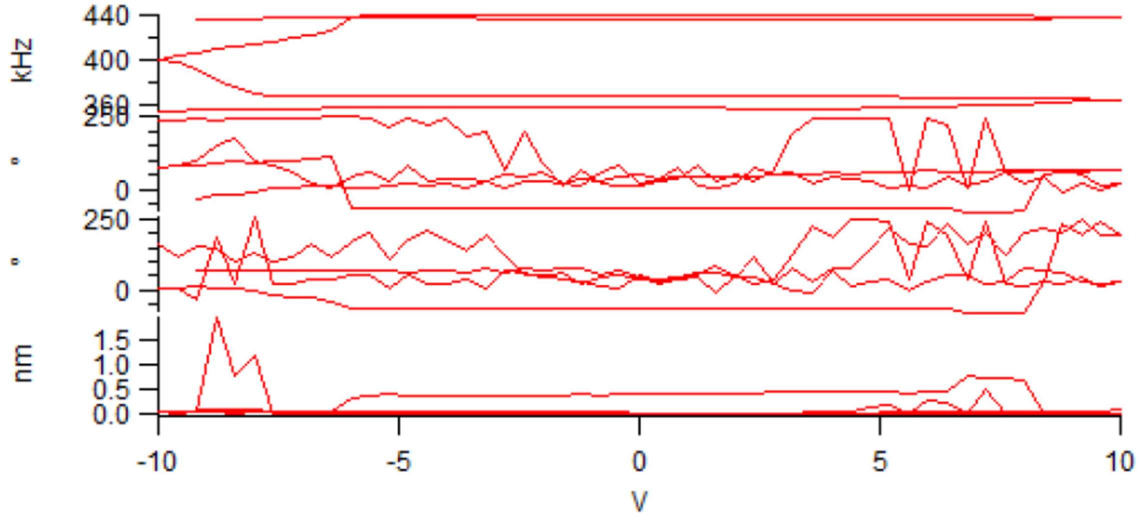

Figure S6. PFM off-field measurement of WO_3 – 500°C thin film indicating no piezoresponse.

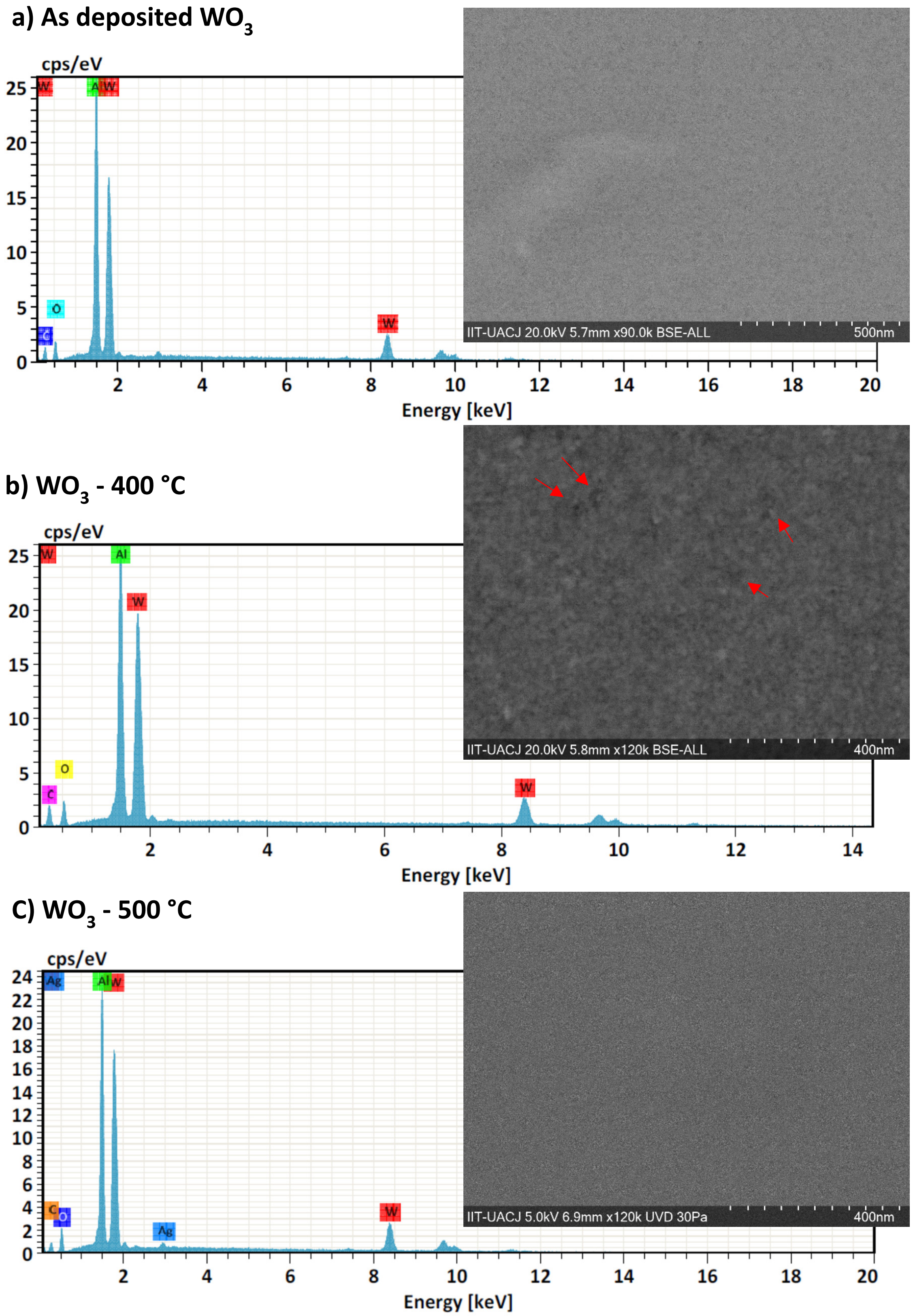

Figure S7. EDS and SEM for WO_3 thin films a) as deposited and annealed at b) 400 °C and c) 500 °C. Red arrows show porosity on the film surface.

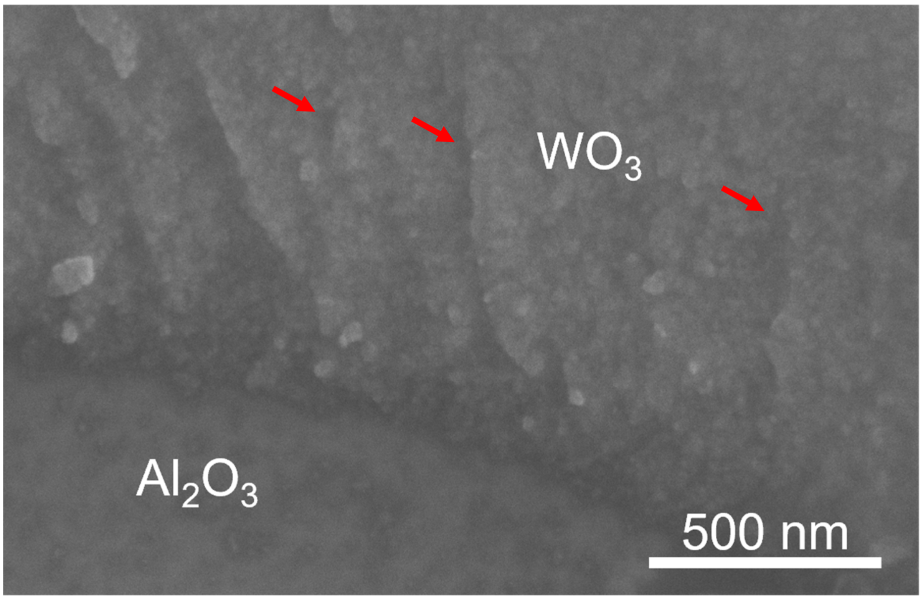

Figure S8. Cross-sectional SEM image of WO_3 thin film annealed at 500 °C where is possible to see porosity within film as indicated by red arrows.

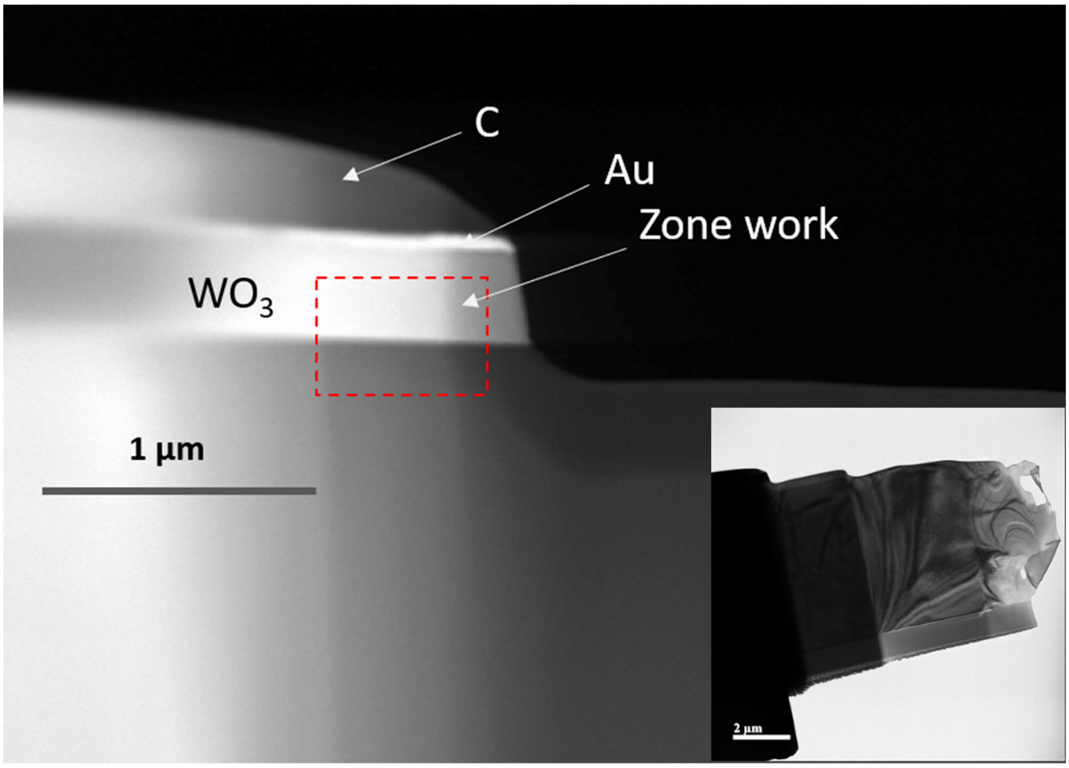

Figure S9. Lamella preparation sequence of images WO_3 – 400 °C thin film for TEM measurements.

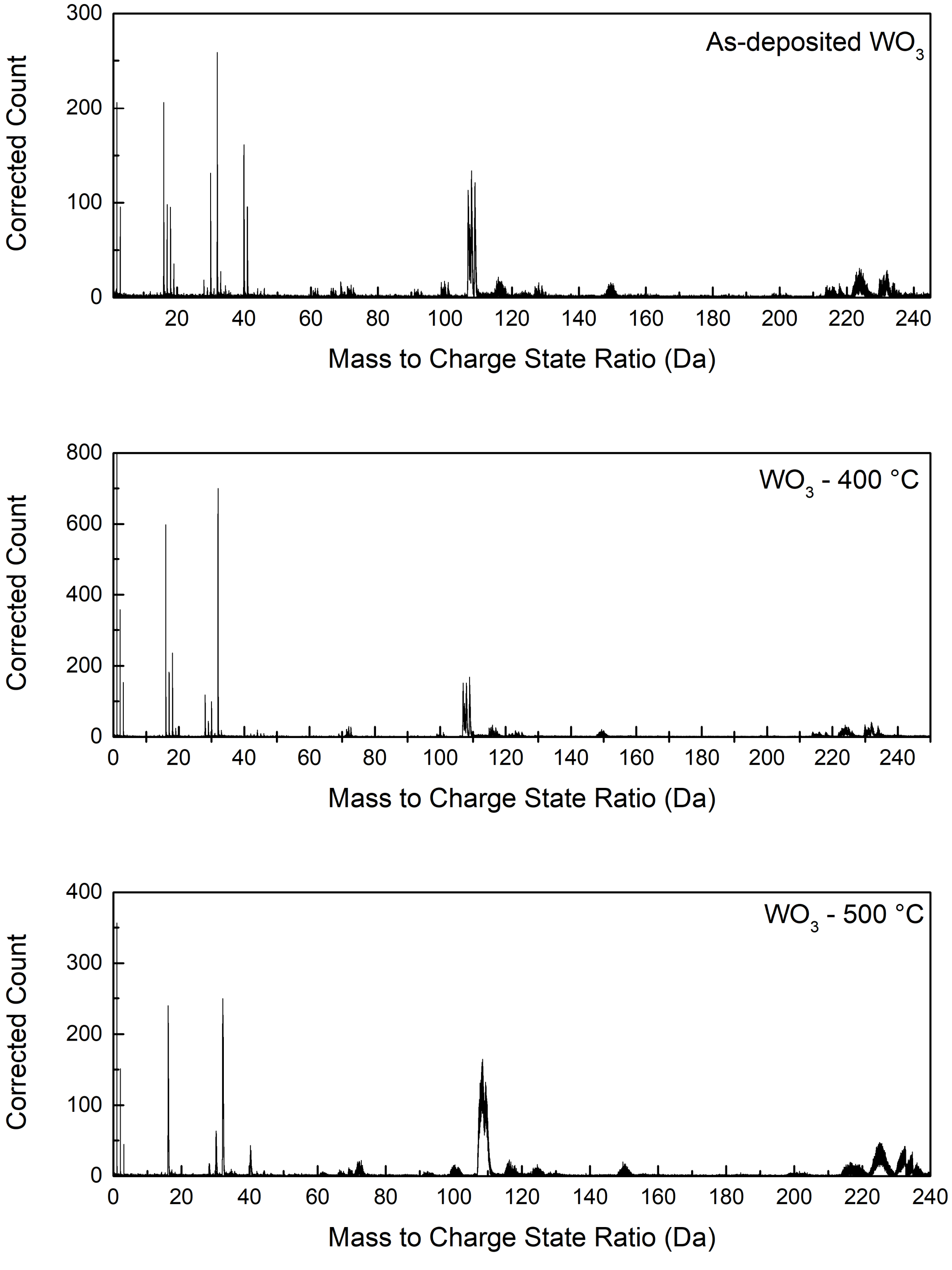

Figure S10. Mass spectra of WO_3 thin films measured by APT, as deposited and processed at 400 °C and 500 °C.
